# Comprehensive single-cell transcriptomic and proteomic analysis reveals NK cell exhaustion and unique tumor cell evolutionary trajectory in non-keratinizing nasopharyngeal carcinoma

**DOI:** 10.1186/s12967-023-04112-8

**Published:** 2023-04-25

**Authors:** Cuimin Chen, Chun Wang, Ruifang Pang, Huanyu Liu, Weihua Yin, Jiakang Chen, Lili Tao

**Affiliations:** 1 Department of Pathology, Peking University Shenzhen Hospital, Shenzhen, China; 2 Department of Precision Research Institute, Peking University Shenzhen Hospital, Shenzhen, China

**Keywords:** Proteomics, single-cell RNA sequencing, NK-NPC, NK cell exhaustion, EBV, Evolutionary trajectory

## Abstract

**Background:**

Nonkeratinizing nasopharyngeal carcinoma (NK-NPC) has a strong association with Epstein-Barr virus (EBV) infection. The role of NK cells and the tumor cell evolutionary trajectory in NK-NPC remain unclear. In this study, we aim to investigate the function of NK cell and the evolutionary trajectory of tumor cells in NK-NPC by single-cell transcriptomic analysis, proteomics and immunohistochemistry.

**Methods:**

NK-NPC (n = 3) and normal nasopharyngeal mucosa cases (n = 3) were collected for proteomic analysis. Single-cell transcriptomic data of NK-NPC (n = 10) and nasopharyngeal lymphatic hyperplasia (NLH, n = 3) were obtained from Gene Expression Omnibus (GSE162025 and GSE150825). Quality control, dimension reduction and clustering were based on Seurat software (v4.0.2) process and batch effects were removed by harmony (v0.1.1) software. Normal cells of nasopharyngeal mucosa and tumor cells of NK-NPC were identified using copykat software (v1.0.8). Cell-cell interactions were explored using CellChat software (v1.4.0). Tumor cell evolutionary trajectory analysis was performed using SCORPIUS software (v1.0.8). Protein and gene function enrichment analyses were performed using clusterProfiler software (v4.2.2).

**Results:**

A total of 161 differentially expressed proteins were obtained between NK-NPC (n = 3) and normal nasopharyngeal mucosa (n = 3) by proteomics (log_2_ fold change > 0.5 and *P* value < 0.05). Most of proteins associated with the nature killer cell mediated cytotoxicity pathway were downregulated in the NK-NPC group. In single cell transcriptomics, we identified three NK cell subsets (NK1-3), among which NK cell exhaustion was identified in the NK3 subset with high ZNF683 expression (a signature of tissue-resident NK cell) in NK-NPC. We demonstrated the presence of this ZNF683 + NK cell subset in NK-NPC but not in NLH. We also performed immunohistochemical experiments with TIGIT and LAG3 to confirm NK cell exhaustion in NK-NPC. Moreover, the trajectory analysis revealed that the evolutionary trajectory of NK-NPC tumor cells was associated with the status of EBV infection (active or latent). The analysis of cell-cell interactions uncovered a complex network of cellular interactions in NK-NPC.

**Conclusions:**

This study revealed that the NK cell exhaustion might be induced by upregulation of inhibitory receptors on the surface of NK cells in NK-NPC. Treatments for the reversal of NK cell exhaustion may be a promising strategy for NK-NPC. Meanwhile, we identified a unique evolutionary trajectory of tumor cells with active status of EBV-infection in NK-NPC for the first time. Our study may provide new immunotherapeutic targets and new sight of evolutionary trajectory involving tumor genesis, development and metastasis in NK-NPC.

**Supplementary Information:**

The online version contains supplementary material available at 10.1186/s12967-023-04112-8.

## Introduction

Nasopharyngeal carcinomas (NPCs) arise in the nasopharyngeal mucosa and show microscopic or ultrastructural evidence of squamous differentiation. There are three histological subtypes of NPCs, including nonkeratinizing NPC (NK-NPC), keratinizing NPC (K-NPC), and basaloid squamous cell carcinoma. NK-NPC is the most common subtype and has a strong association with Epstein-Barr virus (EBV) infection, especially in endemic regions such as southeastern China [1]. Pathologically, NPCs are characterized by heavy infiltration of immune cells around and within the tumor [2], suggesting a remarkably complex tumor microenvironment (TME) [3].

T-cell exhaustion can lead to immunosuppressive microenvironment in tumors. The T-cell exhaustion landscape has been reported in NK-NPC [4]. T-cell based immunotherapy is currently the main method for tumor immunotherapy, but only about 25% of patients respond to the treatment [5], which suggests that there may be other mechanisms in the tumor microenvironment that inhibit the tumor immunity. NK cells play an important role in the innate immune response against tumors. However, the immunological landscape of NK cells has not been reported in NK-NPC before. Besides, many solid tumors have been reported to have unique tumor cell evolutionary trajectories associated with tumor genesis, progression and metastasis. But NK-NPC tumor cell evolutionary trajectories have not been reported in the previous single-cell transcriptomic studies [4, 6, 7].

In this study, we integrated proteomics and single-cell transcriptomics to perform a comprehensive study of NK-NPC. The proteomic analysis revealed that most of proteins associated with NK cell-mediated cytotoxicity were downregulated in NK-NPC group, indicating the functional inhibition of NK cells. In single cell transcriptomics, we identified a subset of tissue-resident NK cells that expressed exhaustion markers in NK-NPC, which have not been reported before. In addition, we revealed for the first time that the evolutionary trajectory of tumor cells in NK-NPC was associated with the status of EBV infection (active or latent). We further found the key cellular interactions leading to immunosuppression in NK-NPC. Our findings revealed the immunological landscape of NK cells and provide potential immunotherapeutic targets for the treatment of NK-NPC.

## Materials and methods

### Data collection

Paraffin sections of NK-NPC (n = 3) and normal nasopharyngeal mucosa (n = 3) were collected for proteomic assays in Peking University Shenzhen Hospital. The diagnoses were confirmed by three experienced pathologists. The NK-NPC single-cell transcriptome sequencing dataset (GSE162025) was obtained from the Gene Expression Omnibus database (https://www.ncbi.nlm.nih.gov/geo/). The NLH single-cell transcriptome sequencing dataset (GSE150825) was obtained from the Gene Expression Omnibus database (https://www.ncbi.nlm.nih.gov/geo/). The study was approved by the Ethical Review Committee of Peking University Shenzhen Hospital (PKUSZH) (approval number: 2022130), and all experiments were performed in accordance with the ethical guidelines of the Declaration of Helsinki.

### Proteomic analysis

A proteomic analysis was performed on the individual FFPE patient samples. In short, proteins were extracted from the deparaffinized biopsies, digested into peptides, and dissolved in 0.1% formic acid for LC-MS analysis. Samples were analyzed on a commercial C18 column (Acclaim PepMap RSLC, 75 μm × 50 cm, Thermo Scientific) using an Ultra3000 UHPLC connected to Exploris 480 mass spectrometer (Thermo Scientific). The generated MS/MS spectra were searched against the Uniprot Human database (https://www.uniprot.org) using the SEQUEST searching engine in the Proteome Discoverer 2.4 software (PD, Thermo Scientific, Waltham, MA, USA). Label-free quantification was carried out only for proteins with two or more unique peptide matches. Protein ratios were calculated as the median of all peptide hits belonging to a protein. Quantitative precision was expressed as the protein ratio variability.

### Immunohistochemical staining (IHC)

Immunohistochemistry was carried out using a Ventana BenchMark ULTRA automated immunostaining system, following the manufacturer’s recommendation (Ventana Medical Systems, Tucson, AZ, USA). The primary antibody for anti-B2M (clone B2M/961, Abcam, Cambridge, MA) was diluted to 1:100. Positivity was defined by more than 10% of tumor cell membranes staining up. Other primary antibodies included CD56 (clone MX039, prediluted, Maixin, Fuzhou, China), granzyme B (clone EP230, prediluted, Maixin, Fuzhou, China), TIGIT (E5Y1W, 1:700, Cell signaling technology, Massachusetts, USA) and LAG3 (D2G4O, 1:200, Cell signaling technology, Massachusetts, USA).

### Differential protein analysis and functional enrichment analysis

Differential protein analysis was performed using ‘limma’ software, GSEA and KEGG enrichment analysis of differential genes were performed using ‘clusterProfiler’ software. Heatmap analysis was performed using ‘pheatmap’ software.

### Single-cell RNA sequencing data processing

In this study, data analysis of single-cell transcriptomics was performed mainly using the R (version 4.1.2). The data were first converted to Seurat objects using the Seurat (v4.0.2) and then initially filtered according to the following requirements: (i) the number of genes detected in a single cell must be more than 200; (ii) the number of genes detected in a single cell must be less than 4000; and (iii) the percentage of mitochondrial genes detected in a single cell must be less than 5%. The number of genes detected less than 200 was considered as broken dead cells, and the number of genes detected more than 4000 was considered as double cells due to cell droplet encapsulation error. Gene expression matrices were generated by log normalization and linear regression using the NormalizeData and ScaleData functions in the Seurat. Principal component analysis was performed using the RunPCA function for dimensional reduction clustering, and TSNE was used to visualize the identified cell subpopulations. Finally, the characteristic gene expression of the cells was visualized using the Seurat software (Dotplot, FeaturePlothe and other functions). Cell types were identified using ‘Single R’ software combined with the interpretation of experienced pathologists.

### Identification of epithelial subtypes

To further explore the tumor cell subpopulations in NK-NPC, all epithelial cells in NK-NPC single cell data were extracted and the process of dimensional reduction and clustering was repeated using Seurat software. The benign and malignant epithelial cells were identified using the copykat software, and the malignant cells were clustered using the copykat software to obtain tumor subtype1 and tumor subtype2 subsets.

### Trajectory analysis

SCORPIUS software was used to characterize the evolutionary trajectories among the 4 clusters cell subtypes obtained from the downscaling clustering of tumor subtype1.

### Cell–cell interaction analysis

CellChat (v1.4.0) software was used to infer, analyze and visualize intercellular communications among various cell subsets in NK-NPC. Seurat normalized expression matrices were used for ligand-receptor interaction analysis. The list of known ligand-receptor pairs was obtained from CellChatDB, a literature-supported database of mouse and human ligand and receptor interactions. We first identified overexpressed ligands or receptors in various cell types and then inferred communication probabilities by calculating all ligand and receptor interactions associated with each signaling pathway.

## Results

### The function of NK cells was inhibited in the NK-NPC

**Fig. 1:**
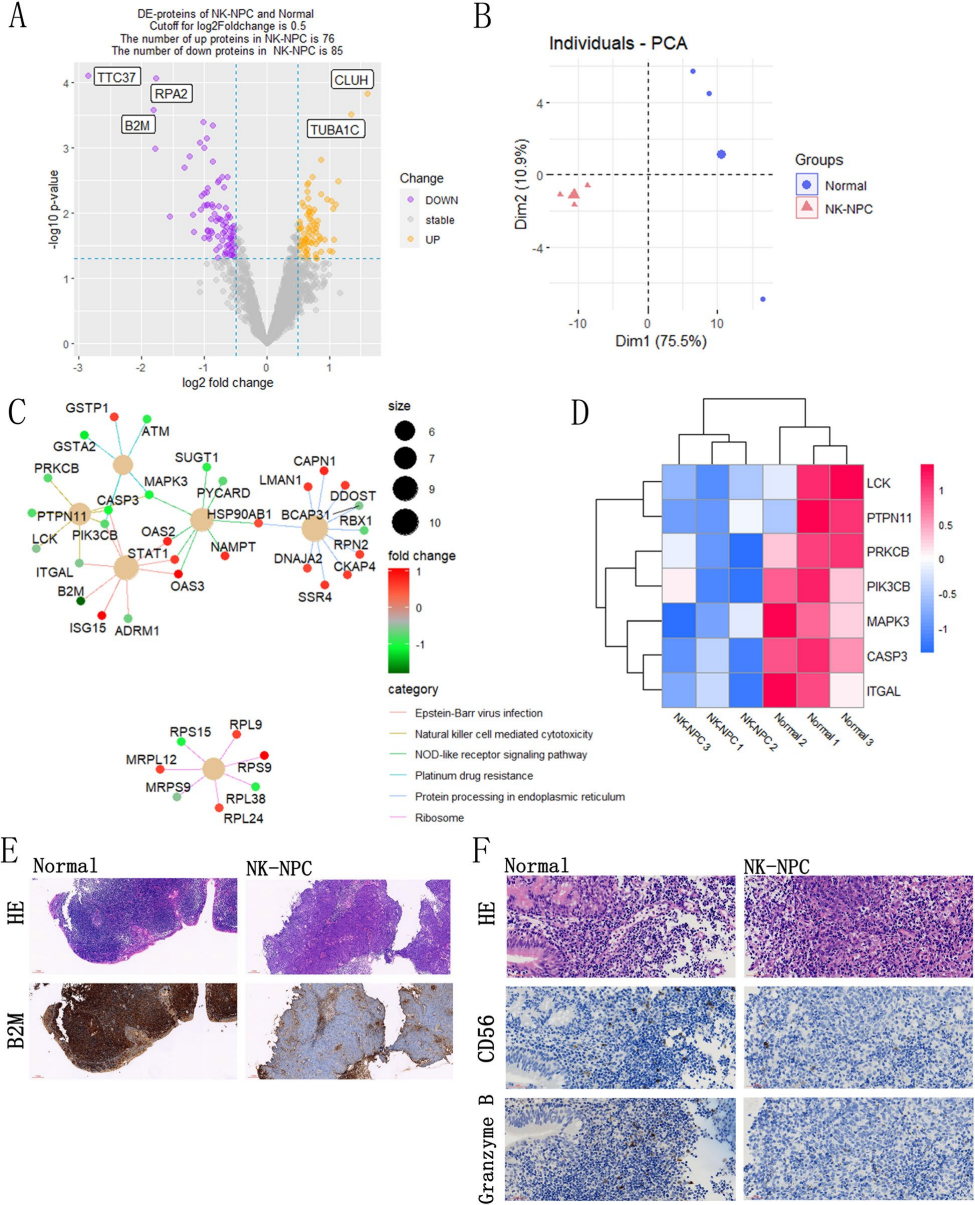
NK-NPC proteomics results. **A** Volcano plot of differentially expressed proteins (DE-proteins). There are 76 proteins upregulated and 85 proteins downregulated in the NK-NPC group compared to the normal group, where proteins with fold change of log2foldchange > 0.5 and *P* value < 0.05 were defined as differentially expressed proteins. **B** The differentially expressed proteins between the 3 NK-NPC and 3 normal nasopharyngeal tissues were well distinguished using Principal Component Analysis (PCA). **C** KEGG enrichment analysis was performed on 161 differentially expressed proteins. **D** Protein heatmap showed that the DE-proteins enriched in the nature killer cell mediated cytotoxicity pathway were significantly downregulated in the NK-NPC group, including PRKCB, PTPN11, LCK, ITGAL, PIK3CB, CASP3 and MAPK3. **E** Representative images of immunohistochemical staining of B2M protein in the normal group and the NK-NPC group. **F** Representative images of immunohistochemical staining of CD56 and granzyme B in the normal group and the NK-NPC group

We performed proteomic analysis of NK-NPC (n = 3) and normal nasopharyngeal mucosa (n = 3), and a total of approximately 3950 proteins were detected (Additional file 1). Only proteins detected in all samples were retained for differential protein expression analysis using limma software, and a total of 161 differentially expressed proteins were obtained (DE-proteins, log2foldchange > 0.5, *P* value < 0.05, Fig. 1A). We identified the top5 significantly regulated proteins which include TTC37, RPA2, B2M, CLUH, TUBA1C. CLUH, TUBA1C are upregulated, while TTC37, RPA2, B2M are downregulated in NK-NPC group. PCA clustering was performed on the 6 samples according to the expression of these 161 differentially expressed proteins, and NK-NPC and normal nasopharyngeal mucosa can be well distinguished by proteomics (Fig. 1B). Functional enrichment analysis of these 161 DE-proteins was conducted (Fig. 1C). We drew the protein heatmap and observed that the DE-proteins (PRKCB, PTPN11, LCK, ITGAL, PIK3CB, MAPK3 and CASP3) associated with NK cell-mediated cytotoxicity were all downregulated in NK-NPC (Fig. 1D), suggesting that the function of NK cells may be inhibited. In the NK-NPC group, proteins related to Natural killer cell mediated cytotoxicity and Platinum-resistance-related proteins were mostly downregulated; Proteins related to protein processing in the endoplasmic reticulum and EBV infection pathway were mostly upregulated. B2M (β2-microglobulin), a non-glycosylated protein, has a molecular mass of 11,800 Da. All nucleated cells could synthesize B2M, which forms an immutable major histocompatibility complex (MHC) class I antigen small light chain subunit through non-covalent binding on the cell surface and maintain the natural conformation of MHC classI. B2M is significantly downregulated in NK-NPC and plays a role in EBV infection, which may be related to the inhibition of MHC classI antigen presentation [8, 9]. We performed immunohistochemistry and confirmed that B2M was significantly downregulated in NK-NPC compared to the normal nasopharyngeal tissue (Fig. 1E). MHC-I has been demonstrated to be an inhibitory receptor of NK cells, and loss of MHC-I on the cell surface due to B2M loss would render cancer cells more vulnerable to NK cells [10]. In addition, we performed immunohistochemistry and found that compared to the normal nasopharyngeal mucosa tissue, the number of CD56 + NK cells and the expression of granzyme B, one of the key cytotoxic effector of NK cell, were decreased in NK-NPC (Fig. 1F).

### Single-cell transcriptome altas of NK-NPC

**Fig. 2:**
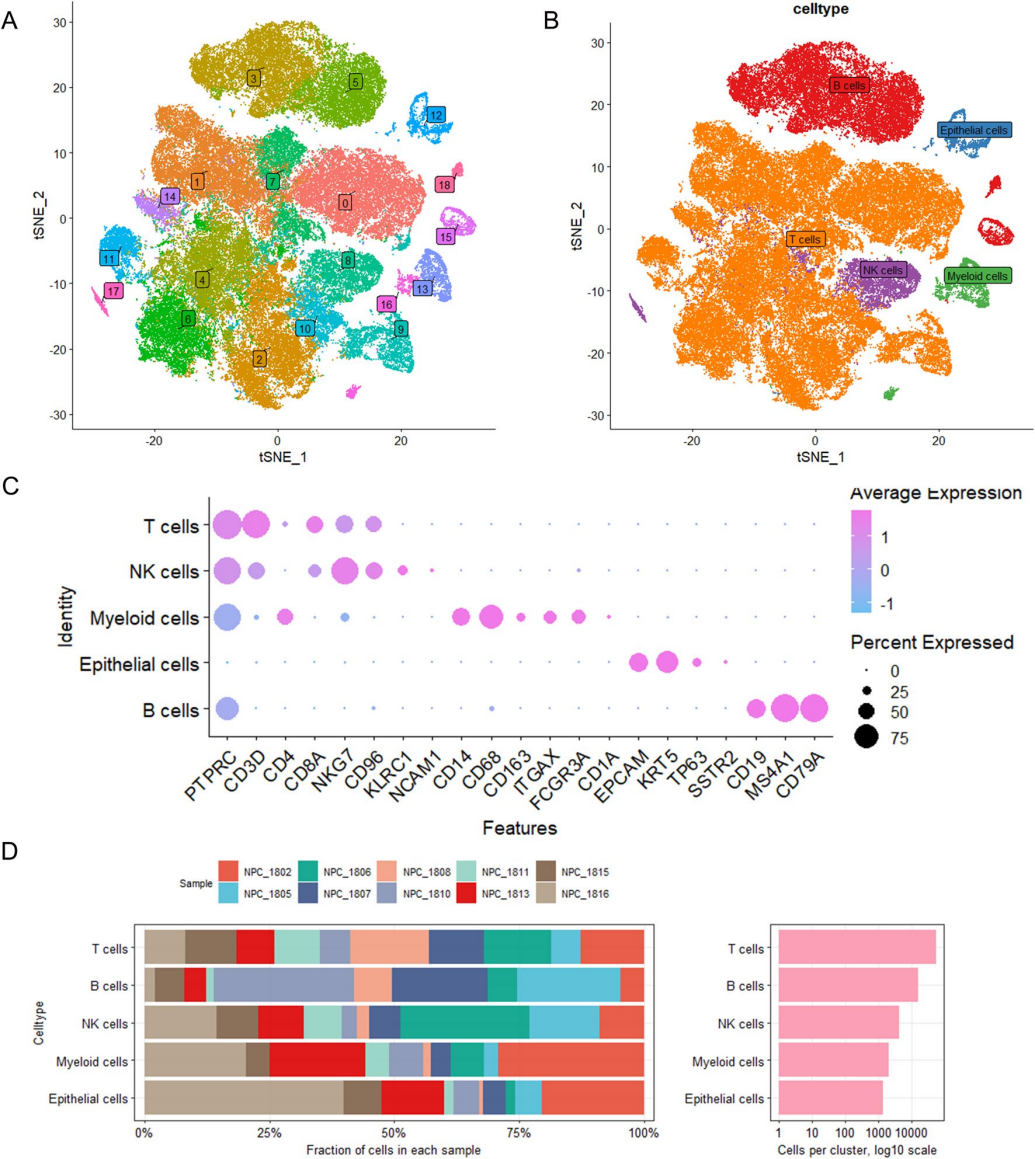
NK-NPC single cell atlas. **A** 19 clusters were obtained by dimension reduction and clustering 79,000 cells. **B** The 19 clusters are identified by their respective marker genes as different types of cells: Epithelial cells, T cells, NK cells, Myeloid cells and B cells. **C** Marker genes of different types of cells. **D** A bar graph of the proportion of cell types in each of the 10 samples

A published single-cell sequencing result of 10 EBV-positive NPC patients were recruited for analysis in this study. After rigorous data quality control and filtering (Additional file 2), a total of about 79,000 cells were obtained. These cells were identified by ‘Seurat’ software with 19 clusters in descending clusters (Fig. 2A). Next, we annotated 19 clusters of cells into 5 cell types using cell-specific markers (Fig. 2B), which included: T cells (PTPRC, CD3D and CD8A), NK cells (PTPRC, NKG7, CD96, KLRC1 and NCAM1), Myeloid cells (CD14, CD68, CD163, ITGAX and FCGR3A), Epithelial cells (EPACM, KRT5, TP63 and SSTR2), and B cells (CD19, MS4A1 and CD79A) (Fig. 2C). We counted the proportion of all the cells from different NK-NPC patients, ensuring that the cells were not unique to any patient. The proportions of different cell types from different patients were inconsistent. T cells, B cells and NK cells rank the top three among all cells. (Fig. 2D). These results suggested obvious tumor heterogeneity in NK-NPC.

### ZNF683 + tissue-resident NK cells are exhausted in NK-NPC

**Fig. 3:**
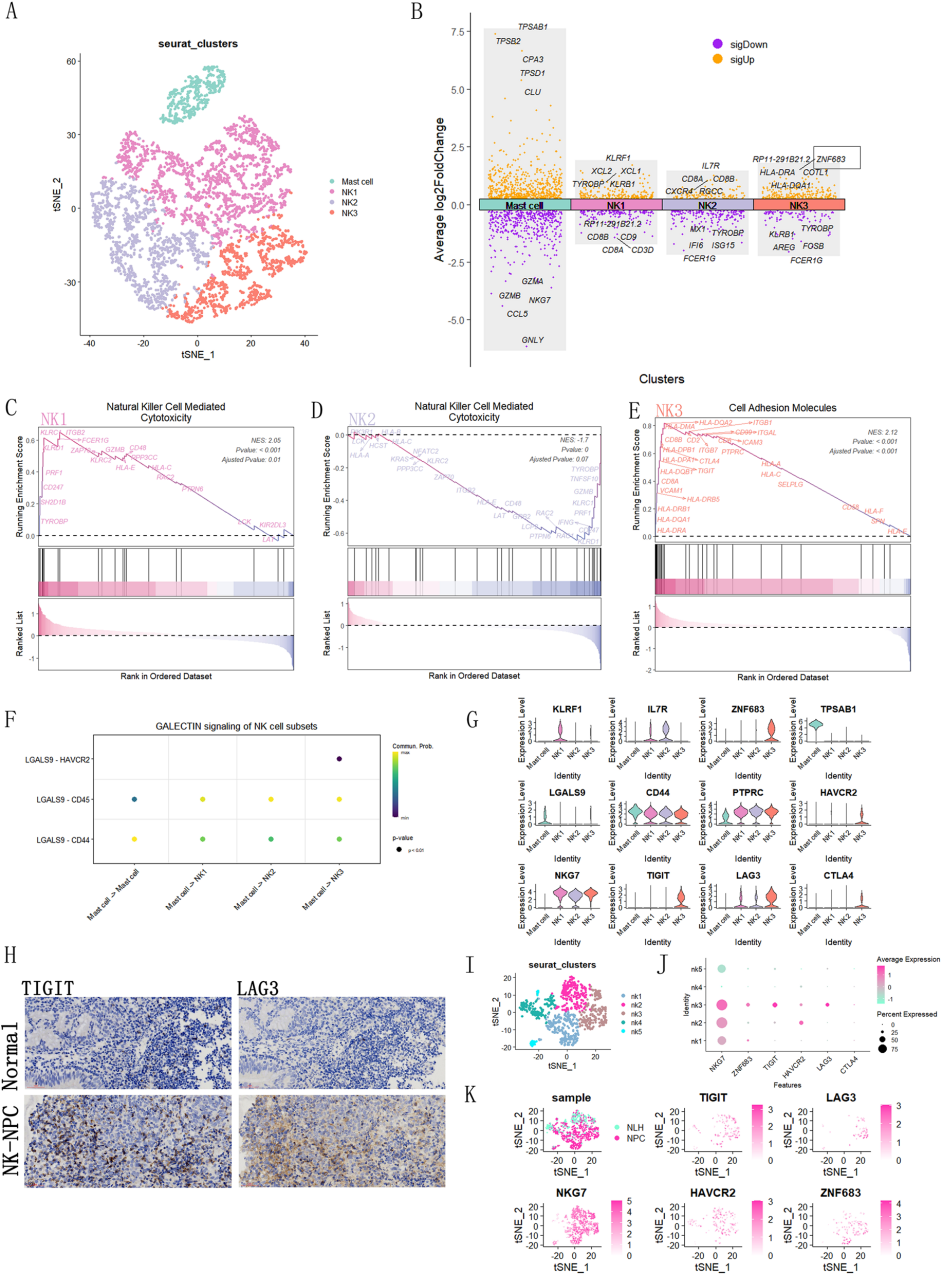
NK cell subtype analysis and exhaustion landscape. **A** NK1-3 cell subsets were obtained by dimension reduction and clustering of NK cells. **B** Marker genes of NK1-3 subsets, sigDown: downregulated genes, sigUp: upregulated genes; The upregulated top5 genes in NK3 subset includes ZNF683. **C** GSEA results showed that nature killer cell-mediated cytotoxicity was activated in the NK1 subset; NES > 0, activated; NES < 0, inhibited. **D** GSEA results showed that nature killer cell- mediated cytotoxicity was inhibited in the NK2 subset. **E** GSEA results showed that cell adhesion molecules was activated in the NK3 subset. **F** Cell-cell interactions between mast cells and NK1-3 subsets. **G** Expression of cell exhaustion markers: HAVCR2, TIGIT, LAG3 and CTLA4 in NK1-3 subsets, with NK3 showing the highest expression of TIGIT and LAG3, and partial expression of HAVCR2 and CTLA4. Besides, NK3 showed the highest expression of ZNF683. **H** Representative images of immunohistochemical staining of TIGIT and LAG3 in the normal nasopharyngeal mucosa tissue and NK-NPC tissue. **I** NK cells in the GSE150825 dataset were downscaled and clustered into five cell subsets, nk1-5. **J** The expression of NKG7, ZNF683, TIGIT, HAVCR2, LAG3 and CTLA4 in the nk1-5 subsets, in which a relatively high expression of ZNF683, TIGIT and LAG3 was observed in the nk3 subset in NK-NPC. **K** The expression of TIGIT, LAG3, NKG7, HAVCR2 and ZNF683 were mapped to nk1-5 subsets, and TIGIT, LAG3 and ZNF683 were mainly expressed in the NK-NPC group, while they were less expressed in the NLH group

In the proteomic results, we found that the differential proteins associated with natural killer cell mediated cytotoxicity were all downregulated in the tumor group. To better investigate the role of NK cells in NK-NPC, we used ‘Seurat’ software to downscale the NK cells in the NK-NPC single cell transcriptional profile and obtained three NK cell subsets (NK1-NK3, Fig. 3A), where the mast cells were probably mixed in the NK cell subsets because of the low resolution of the first downscale clustering. We used the ‘FindAllMarkers’ function to find the cellular signature genes of the three NK cell subsets, and used the scRNAtoolVis software to map the volcanoes of the signature genes and show the top 5 upregulated and downregulated genes in the respective cell subsets (Fig. 3B). ZNF683 was the most significantly differential gene between the NK3 subset and other cell subsets, suggesting that the NK3 subset may be a tissue-resident NK cell [11]. We performed GSEA enrichment analysis of the marker genes of NK1-NK3 subsets separately (Fig. 3C and E, for all enrichment analysis results, see Additional file 3). NK cell-mediated cytotoxicity was activated in the NK1 subset (NES = 2.06, *p* < 0.001, Fig. 3C), NK cell-mediated cytotoxicity was inhibited in the NK2 subset (NES=− 1.7, *p* < 0.05, Fig. 3D), and no pathway associated with NK cell-mediated cytotoxicity was detected in the NK3 subset. Besides, marker genes of the NK3 subset and immune cell exhaustion-related markers, including TIGIT and CTLA4, promoted the cell adhesion molecular pathway (NES = 2.14, *p* < 0.001, Fig. 3E). We performed intercellular communication among NK1-NK3 subsets and mast cells (Fig. 3F, Intercellular communication between all celltypes is shown in Additional file 4). The interaction of LGALS9-CD44, LGALS9-CD45 and LGALS9-HAVCR2 between mast cells and NK1-3 subsets may impair the function of NK cell [12]. Both proteomics and single-cell transcriptomics suggested that NK cell-mediated cytotoxicity appeared to be inhibited. We therefore examined the expression of four cellular exhaustion markers, HAVCR2, TIGIT, LAG3 and CTLA4, in NK cells (Fig. 3G) and found that TIGIT was uniquely overexpressed in the NK3 subpopulation, while LAG3 was significantly upregulated in the NK3 subpopulation and CTLA4 and HAVCR2 were partially upregulated in NK3. Taken together, the NK3 subset may be a population of tissue-resident NK cells, and the NK3 subpopulation may be the predominant exhausted NK cell type and may have lost its NK cell-mediated cytotoxic function. The NK2 subset may be an NK cell type whose cytotoxicity was inhibited but not yet exhausted, the NK1 subset may be an effector NK cell type that has not yet matured or was not affected by exhaustion. Further, we performed immunohistochemical analysis and observed that compared with the normal nasopharyngeal tissue, the expression of TIGIT and LAG3 was higher in the NK-NPC tissue, indicating that the NK cell function was inhibited or exhausted in NK-NPC (Fig. 3H). It is worth noting that the inhibition of NK cell-mediated cytotoxicity can be reversed, whereas the terminal exhaustion of NK cells is irreversible; Mast cells may play a role in regulating NK cell exhaustion, and how to restore the inhibited NK cell function and reverse the exhaustion of NK cells may be a promising immunotherapeutic target for NK-NPC. To confirm that the exhaustion of NK cells was observed only in tumors on the single-cell data level, we performed the same analytic procedure as above for a dimensionality reduction clustering of NK cells in the NLH (Additional file 5). Five subsets were obtained by downscaling the NK cells of GSE150825(nk1-5, Fig. 3I), and compared to the NLH group, higher expression of ZNF683, TIGIT, and LAG3 could be observed in the nk3 cell subset (Fig. 3J) in the NK-NPC group, which is consistent with our previous results. We also found that the expression of TIGIT, LAG3 and ZNF683 was mainly concentrated in the NK-NPC group rather than the NLH group by mapping the expression of NKG7, TIGIT, LAG3, HAVCR2 and ZNF683 to the tSNE results of the nk1-5 cell subsets (Fig. 3K).

### NK-NPC tumor cells might induce NK cell exhaustion through LGALS9-CD44

**Fig. 4:**
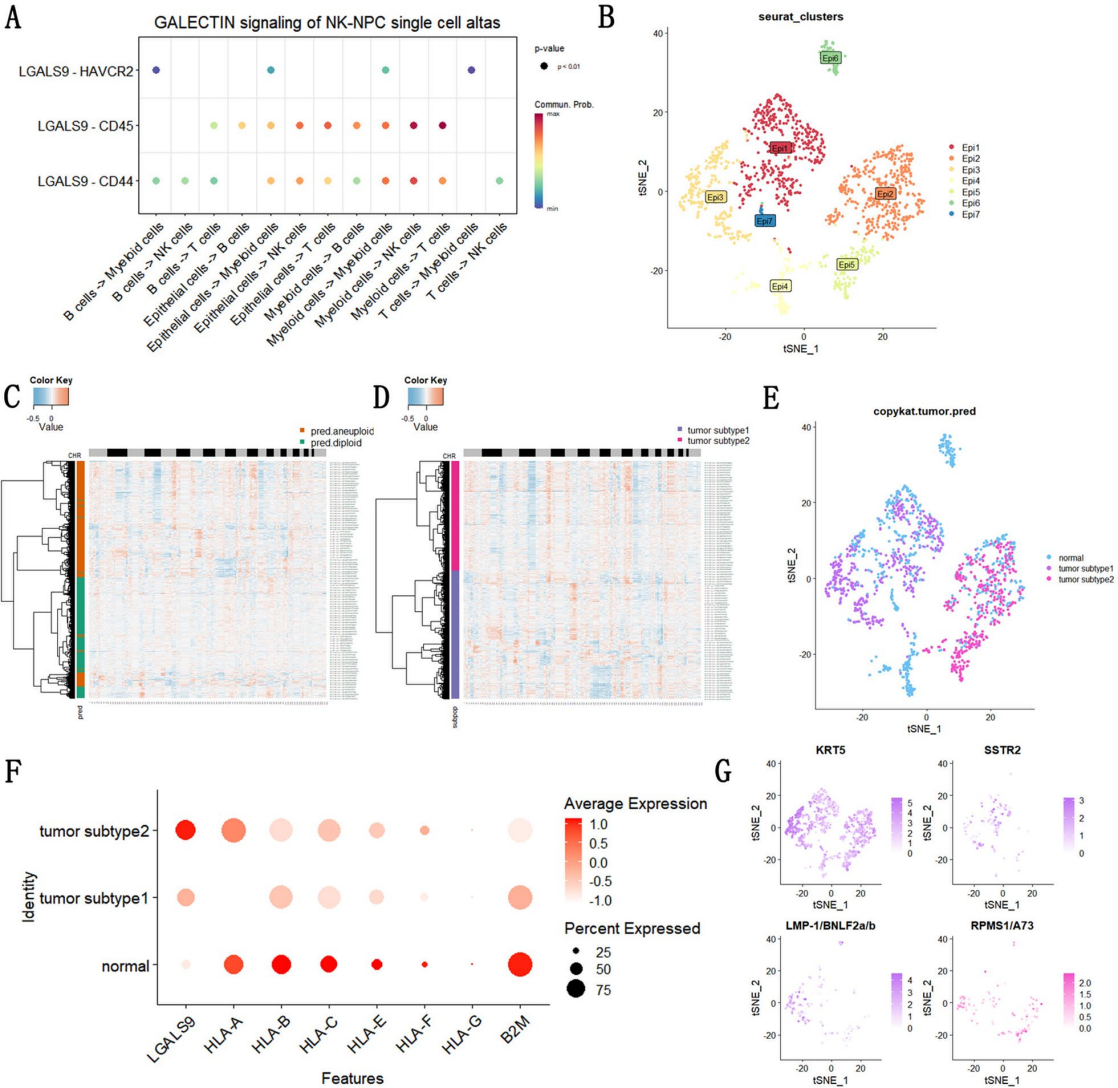
Analysis of NK-NPC tumor cell subtypes and cellular evolutionary trajectories. **A** Cell-cell interactions between all cell types about GALECTIN signaling. **B** Epi1-7 cell subsets were obtained by reduced-dimensional and clustering of Epithelial cells. **C** The copykat software identifies the benign and malignant epithelial cells, with orange representing copy number increase, blue representing copy number deletion, and gray and black interspersed above representing different chromosomes; pred.aneuploid: malignant; pred.diploid: benign. **D** The copykat software further identifies malignant tumor cells into two subtypes, tumor subtype1 and tumor subtype2. **E** The two subtypes, tumor subtype1 and tumor subtype2, were mapped in the TNSE graph. **F** Expression of LGALS9 and MHC class I molecules in tumor subtype1 and tumor subtype2. **G** Expression and distribution of KRT5, SSTR2, LMP-1/BNLF2a/b and RPMS1/A73 in tumor subtype1 and tumor subtype2

To explore other factors that may induce NK cell exhaustion in NK-NPC, we performed a global intercellular communication assay of NK-NPC single cell profiles (Fig. 4A), where an LGALS9-CD44 and LGALS9-CD45 intercellular interaction mode existed between NK cells and multiple cell types, including epithelial cells, T cells, B cells and myeloid cells. T cell exhaustion was also reported in NK-NPC, which is consistent with our findings. We speculated that NK cells may be induced to exhaust in a similar manner in NK-NPC. The ability of myeloid cells to inhibit the function of NK cells has been reported in many studies, however, the interaction between epithelial cells and NK cells through LGALS9-CD44 and LGALS9-CD45 has not been reported before, which aroused our interest. We downscaled the epithelial cells in the NK-NPC single-cell atlas by ‘Seurat’ software to obtain seven epithelial cell subsets (Epi1-Epi7, Fig. 4B), and subsequently we identified the benign (pred.diploid) and malignant (pred.aneuploid) epithelial cells using the copykat software (Fig. 4C). The malignant epithelial cells were again clustered into two tumor cell subtypes (Fig. 4D) by copykat software, namely tumor subtype1 and tumor subtype2, and mapped to the TSNE results of the Epi1-Epi7 subsets (Fig. 4E). NK cells can recognize and kill tumor cells by downregulating MHC class I molecules. Therefore, we also compared the expression of MHC class I in tumor subtype1, tumor subtype2 and normal cells, including HLA-A, HLA-B, HLA-C, HLA-E, HLA-F, HLA-G and B2M (Fig. 4F). Similar to the findings in proteomics and immunohistochemistry, the expressions of MHC class I and B2M were mostly significantly downregulated in tumor cells. LGALS9 was expressed in both tumor subtype1 and tumor subtype2, but was more highly expressed in tumor subtype2, suggesting that tumor subtype2 may be the tumor epithelial cell type that primarily induced NK cell exhaustion. We examined the expression of transcripts encoding EBV proteins (LMP1-BNLF2a/b and RPMS1/A73) in tumor subtype1 and tumor subtype2 (Fig. 4G) to determine whether EBV was latent or active in these tumor cells. Besides, our previous study demonstrated that SSTR2 has a unique immunohistochemical landscape in NK-NPC and that SSTR2 expression has an important role in the diagnosis of nasopharyngeal carcinoma in clinicopathology [13]. Therefore, we also examined the expression of SSTR2 and KRT5 in tumor subtype1 and tumor subtype2 (Fig. 4G). SSTR2 and LMP1-BNLF2a/b were mainly expressed in tumor subtype1, while RPMS1/A73 was mainly expressed in tumor subtype2, suggesting that EBV may be active in tumor subtype1 while be latent in tumor subtype2. We proposed that EBV in its latent state in tumor subtype2 evaded the recognition by CD8 + T cells by downregulating B2M to affect antigen presenting by MHC class I molecules, tumor subtype2 achieved immune escape by continuously expressing LGALS9 to induce functional impairment of T and NK cells, thereby evading recognition and killing by NK cells due to downregulation of MHC class I molecules.

### The cellular evolutionary trajectory of NK-NPC tumor subtype1

**Fig. 5:**
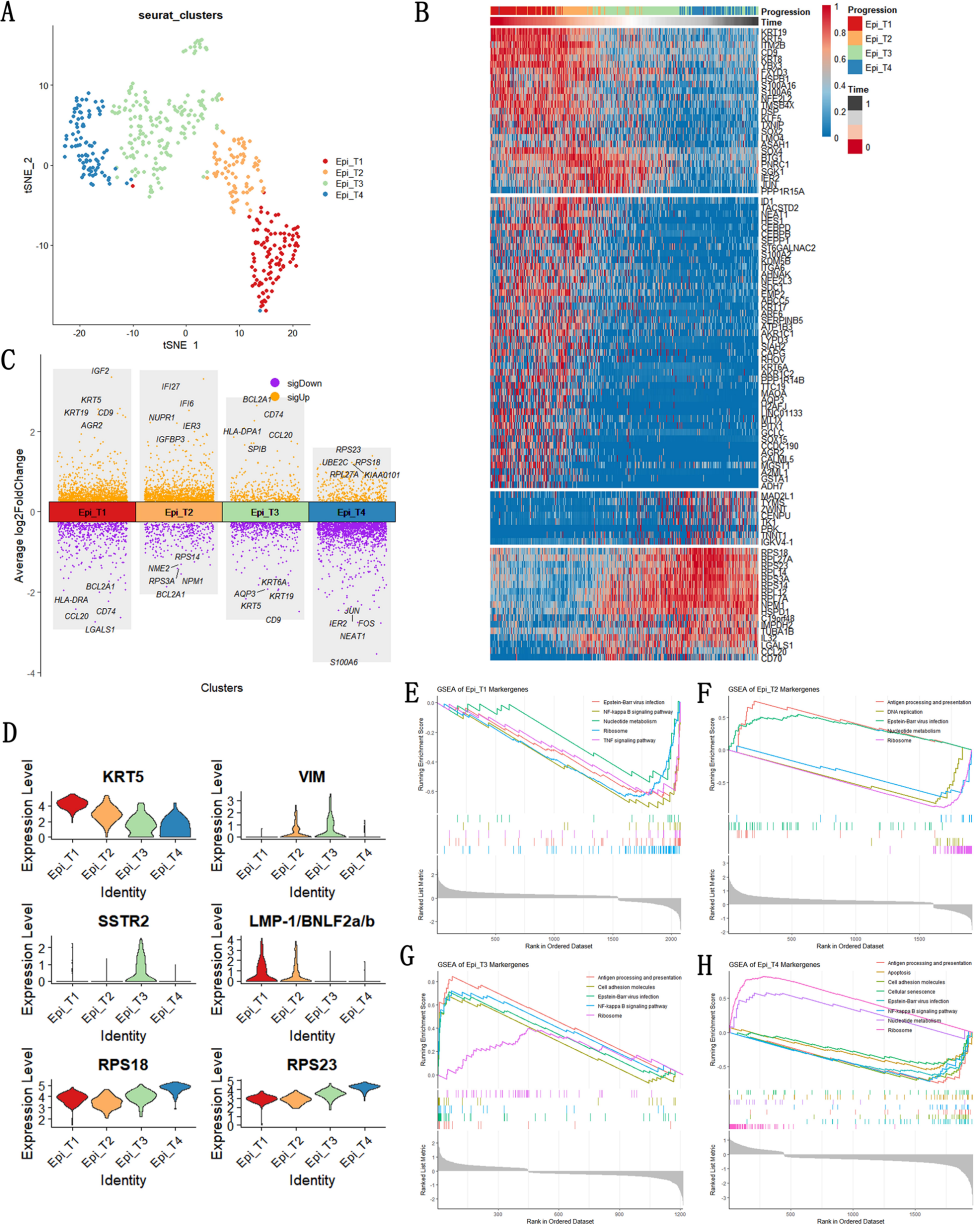
Tumor subtype1 cell evolutionary trajectory and functional enrichment analysis. **A** Epi T1-4 cell subsets were obtained by dimension reduction and clustering of tumor subtype1 subsets. **B** The cellular evolutionary trajectory of tumor subtype1: Time from 0 to 1, epithelial cell evolutionary trajectory direction from Epi T1-Epi T4, corresponding to global gene expression changes. Red is high expression and blue is low expression. **C** Marker genes of Epi T1-4 subsets. sigDown, downregulated genes; sigUp, upregulated genes. **D** Violin plots showing the expression of KRT5, VIM, SSTR2, LMP-1/BNLF2a/b, RPS18 and RPS23 genes in Epi T1-T4 subsets and the variation changes. **E–H** GSEA enrichment analysis of the respective marker genes of Epi T1-T4 subsets

To further investigate the role of EBV activity status in NK-NPC, we downscaled tumor subtype1 by Seurat software and obtained four subsets (Fig. 5A), which were analyzed using SCORPIUS software and found that these four subsets may have spatial and temporal evolutionary trajectories (Fig. 5B). The four subsets were defined as Epi-T1, Epi-T2, Epi-T3 and Epi-T4 according to their spatial and temporal evolutionary trajectories (Fig. 5B). The FindAllMarkers function was used to find cellular signature genes in four cell subpopulations, and the scRNAtoolVis software was used to map the volcanoes of signature genes and show the top5 most significantly up and downregulated genes in the respective cell subset (Fig. 5C). In the global genetic alteration of the evolutionary trajectory (Fig. 5B), we observed an evolution from Epi-T1 to Epi-T4 accompanied by a progressive downregulation of KRT5 (Fig. 5B), suggesting that the process was accompanied by a possible epithelial mesenchymal transition. In addition, the progressive upregulation of ribosomal genes (RPS18, RPL27A, RPS23 and RPL14, etc.) (Fig. 5B) was consistent with the findings in proteomics. The alterative tendency of ribosomal genes was closely associated with EBV infection and the evolultion of tumor cells. In the violin plot we observed a progressive downregulation of KRT5 and progressive upregulations of RPS18 and RPS23 in Epi-T1-T4. Meanwhile, the expression of vimentin in Epi-T2 and Epi-T3, a unique expression of SSTR2 in Epi-T3 and LMP-1/BNLF2a/b in Epi-T1 and Epi-T2 were observed (Fig. 5D). These results suggested that EBV was active in early tumor subtype 1 and affected host cells by encoding LMP-1/BNLF2a/b proteins, and that the process of epithelial mesenchymal transition was in accord with our hypothesis. Further, we performed GSEA enrichment analysis on the respective marker genes of Epi-T1, Epi-T2, Epi-T3 and Epi-T4. In Epi-T1, the EBV infection pathway, the ribosome pathway and the nucleotide metabolism pathway were inhibited (Fig. 5E), and we speculate that during the early stages of EBV infection of nasopharyngeal epithelial cells, the cells are resistant to EBV. As EBV infection progressed, the resistance was diminished. By progression to Epi-T2, the EBV infection pathway was activated, as were the antigen processing and presenting pathways, while the DNA shearing pathway and the nucleotide metabolism and ribosome pathways remained inhibited (Fig. 5F). By progression to Epi-T3, the EBV infection pathway, the antigen processing and presenting pathway, the cell adhesion molecule pathway, the NF-κB signaling pathway and ribosome pathway were activated (Fig. 5G). By progression to Epi-T4, the antigen processing and presenting pathway, the apoptotic pathway, the cellular senescence pathway, the cell adhesion molecule pathway, the NF-κB signaling pathway and the EBV infection pathway were inhibited, while the nucleotide metabolism pathway and the ribosome pathway were activated, especially the ribosome pathway was activated more significantly than in the above three periods (Fig. 5H).

## Discussion

Our research may shed a new light on the study of NK-NPC. Proteomic analysis revealed that proteins associated with NK cell mediated cytotoxicity were downregulated in the NK-NPC group, suggesting that NK cell function was suppressed. Subsequent single cell mapping of NK cell subsets showed that markers involving NK cell inhibition and exhaustion were upregulated in NK-NPC. In addition, we identified a NK cell subset with tissue-resident characteristics, which highly expressed ZNF683. Meanwhile, it was the main NK cell subtype that exhibited the features of exhaustion, with the highest expression of TIGIT and LAG3. TIGIT and LAG3 are classical markers involving immune cell exhaustion [14, 15]. Besides, single-cell transcriptomic atlas revealed a uniqueevolutionary trajectory of tumor cells and a complex network of cellular interactions in NK-NPC.

In recent years, the function of NK cell in tumor immunity has attracted great attention. NK cells play an important role in the innate immune response against tumors. They can recognize and destroy tumor cells through receptor-ligand interaction. However, tumor cells can send negative signals that bind to the inhibitory receptors on the surface of NK cells, thereby mediating functional inhibition or exhaustion of NK cells. Strategies to modulate NK cell activity are emerging as promising tools for the treatment of tumors that showed no clinical response to T cell-based immunotherapy [16]. Single-cell transcriptome analysis revealed three NK cell subsets, among which the tissue resident NK cell subset showed increased expressions of inhibitory receptors, including TIGIT, LAG3 and TIM-3 (HAVCR2) [14, 15], and we proved our conclusion in another single-cell dataset (GSE150825) including NK-NPC and NLH. These inhibitory receptors are associated with NK cell exhaustion. TIGIT and LAG3 were associated with the functional exhaustion of T cell as well [17]. Meanwhile, there were no pathways associated with NK cell-mediated cytotoxicity in the NK3 subset. Further immunohistochemistry showed that compared with the normal nasopharyngeal mucosa tissue, the expression of CD56 (the marker of NK cells) and granzyme B (one of the key cytotoxic effectors of NK cell) decreased, while the expression of TIGIT and LAG3 increased in the NK-NPC tissue, indicating that the NK mediated cytotoxic signaling pathway was inhibited. We speculated that the ligands of NPC tumor cells may bind to various inhibitory receptors of NK cells, such as TIGIT, LAG3 and TIM-3, and reduce the cytotoxicity of NK cells. The functional exhaustion of NK cells leaded to the loss of anti-tumor effects and tumor progression. In recent years, some studies have revealed that reduced NK cell activity was associated with cancer development. Immunotherapy targeting checkpoint inhibitory receptors of NK cell may be a new therapeutic strategy [18]. Notably, the TIGIT receptors are expressed on both T cells and NK cells and associated with exhaustion of these two immune cells [19]. Some experiments showed that antibody targeting TIGIT could reverse the exhaustion of both NK cells and T cells [16]. Clinical trials of TIGIT, LAG3 and TIM-3 monoclonal antibodies as single agents or in combination with PDL1 antibody for the treatment of solid tumors are currently ongoing [18]. Our study showed that NK cell inhibitory receptors such as TIGIT, LAG3 and TIM-3 were all upregulated and that exhaustion of NK cell may induce immune escape and tumor progression in NK-NPC.

In addition, proteomics and immunohistochemical analysis revealed a significant downregulation of B2M in NK-NPC tumor cells, which was associated with EBV infection. The reduction of B2M in tumor cells may lead to the reduction of MHC class I, thus avoiding the immune surveillance of T cells. On the other hand, the reduction of MHC class I normally triggered NK cells to eliminate the tumor cells via “missing-self” recognition. However, NK cell activity was impaired in NK-NPC. Ultimately, tumor cells escape the immune attacks of both the T cells and NK cells. Therefore, immunotherapy targeting NK cell may also restore the NK cell-dependent immune response to NK-NPC tumor cells with decreased MHC class I expression.

Previous studies have shown that EBV-infected NPCs released large amounts of exosomes containing LGALS9 (Galectin-9), which can interact with TIM-3 and induce apoptosis of TIM-3-expressing Th1 cells, leading to the escape of tumor cells from immune recognition [20]. Another study showed that the expression of Galectin9 was higher in the recurrent NPC tumor cells than in the primary NPC cells. The interaction between Galectin-9 and TIM-3 + lymphocytes may mediate the immune escape of NPCs [21]. It has also been reported that LGALS9 can downregulate genes of the NK cell-mediated cytotoxic pathway and impair the function of NK cells [12]. We speculated that, tumor cells can suppress T-cell and NK cell function by secreting LGALS9. The exact mechanism is still unclear and needs to be further investigated by biological experiments.

Many solid tumors have been reported to have unique tumor cell evolutionary trajectories associated with tumor genesis, progression and metastasis. But NK-NPC tumor cell evolutionary trajectories have not been reported in the previous single-cell transcriptomic studies. We identified a distinct evolutionary trajectory in the tumor cell type that were detectable of the EBV transcript LMP-1/BNLF2a/b in NK-NPC. LMP-1 predominantly expressed in early EBV infection. Induced expression of LMP1 during the viral lysis cycle plays a key role in virus production [22]. In our study, higher expression of LMP-1/BNLF2a/b was detected in tumor subtype1 compared to tumor subtype2, indicating that EBV was active in tumor subtype1. Cellular evolutionary trajectory analysis revealed a distinct cellular evolutionary trajectory of tumor cells in the EBV-active state of NK-NPC, while this unique cellular evolutionary trajectory was not found in tumor subtype 2 which LMP-1/BNLF2a/b was low expressed. We depict evolutionary trajectory of NK-NPC tumor cells for the first time, the biomarker we detected in different stage of tumor progression might be helpful for tumor staging in clinicopathology diagnosis.

## Conclusion

In conclusion, the present study revealed the NK cell exhaustion landscape in NK-NPC. The tumor cells may induce the functional inhibition and exhaustion of NK cell by producing negative ligands that bind to the inhibitory receptors on the surface of NK cells. Besides, the tumor cells may inhibit the function of NK cells by the expression of LGALS9 in NK-NPC. This study further identified a unique evolutionary trajectory of tumor cells in the EBV-active status in NK-NPC for the first time. Our findings provide potential therapeutic targets and possible biomarkers for tumor surveillance in different stage, thus improving the survival of NK-NPC patients. However, further functional experiments are needed to investigate the potential mechanisms.

## Supplementary Information

**Additional file 1.** NK-NPC proteomic results

**Additional file 2.** GSE162025 data quality control and filtering

**Additional file 3.** All enrichment analysis results of NK1-3

**Additional file 4.** Intercellular communication between all celltypes

**Additional file 5.** Datapreprocessing and quality control process for GSE150825

## Data Availability

The datasets generated during the current study are available in the GEO database (https://www.ncbi.nlm.nih.gov/geo/query/acc.cgi?acc=GSE162025 and https://www.ncbi.nlm.nih.gov/geo/query/acc.cgi?&acc=GSE150825). The list of proteomics results is available in Additional file 1.

## Abbreviations

NK-NPC: Nonkeratinizing nasopharyngeal carcinoma
EBV: Epstein-Barr virus
TME: Tumor microenvironment
FFPE: Formalin fixed paraffin embedding
IHC: Immunohistochemical staining
PCA: Principal component analysis
GSEA: Gene set enrichment analysis
KEGG: Kyoto encyclopedia of genes and genomes
TSNE: T-Distributed stochastic neighbor embedding
DE-proteins: Differentially expressed proteins
MHC class I: Major histocompatibility complex class I
NES: Normalized Enrichment Score
