# Supplementary figures and images for: Comprehensive single-cell transcriptomic and proteomic analysis reveals NK cell exhaustion and unique tumor cell evolutionary trajectory in non-keratinizing nasopharyngeal carcinoma
Source: J Transl Med. 2023 Apr 25;21:278. doi: 10.1186/s12967-023-04112-8 (PMC10127506; doi:10.1186/s12967-023-04112-8)

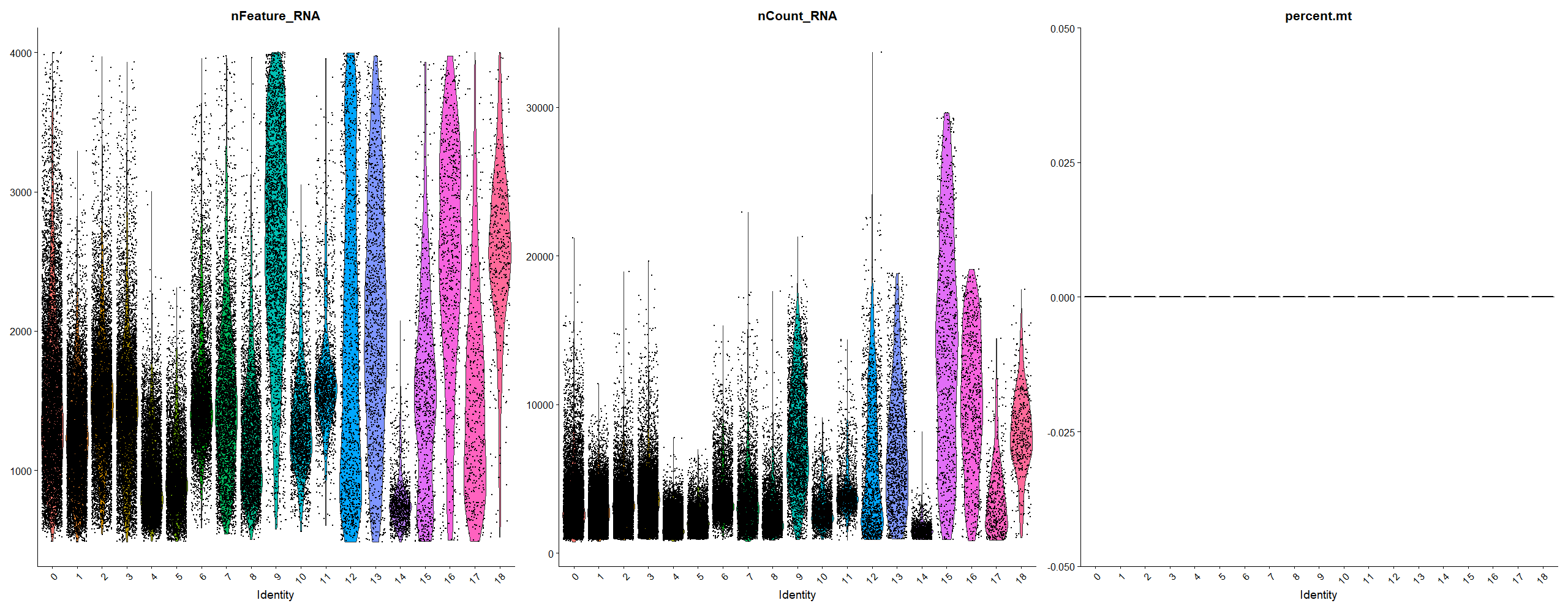

Supplement: Supplementary file 2 — Additional file 2. GSE162025 data quality control and filtering [file 12967_2023_4112_MOESM2_ESM.tiff]

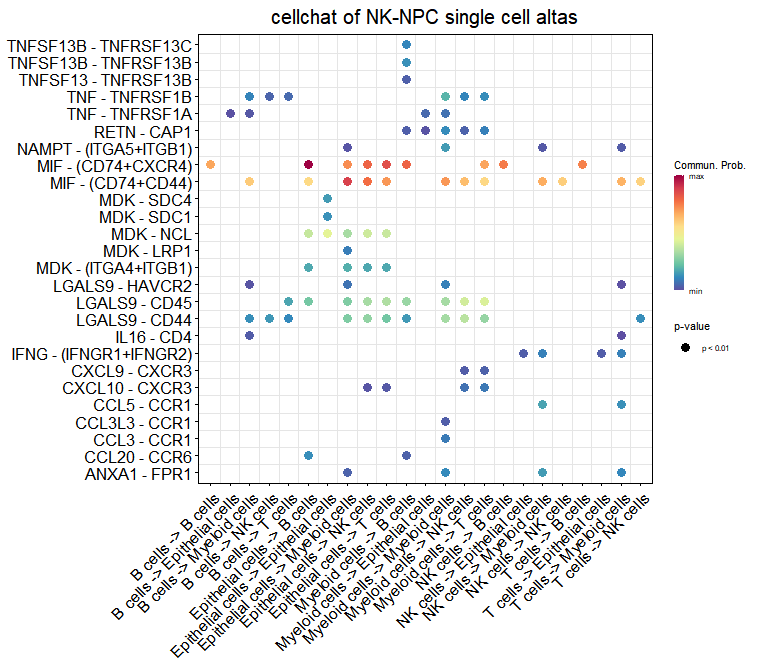

Supplement: Supplementary file 4 — Additional file 4. Intercellular communication between all celltypes [file 12967_2023_4112_MOESM4_ESM.tiff]
