# Supplementary material for: Comprehensive single-cell transcriptomic and proteomic analysis reveals NK cell exhaustion and unique tumor cell evolutionary trajectory in non-keratinizing nasopharyngeal carcinoma
Source: J Transl Med. 2023 Apr 25;21:278. doi: 10.1186/s12967-023-04112-8 (PMC10127506; doi:10.1186/s12967-023-04112-8)

Data preprocessing and quality control process for GSE150825

1、Data cleaning and quality control


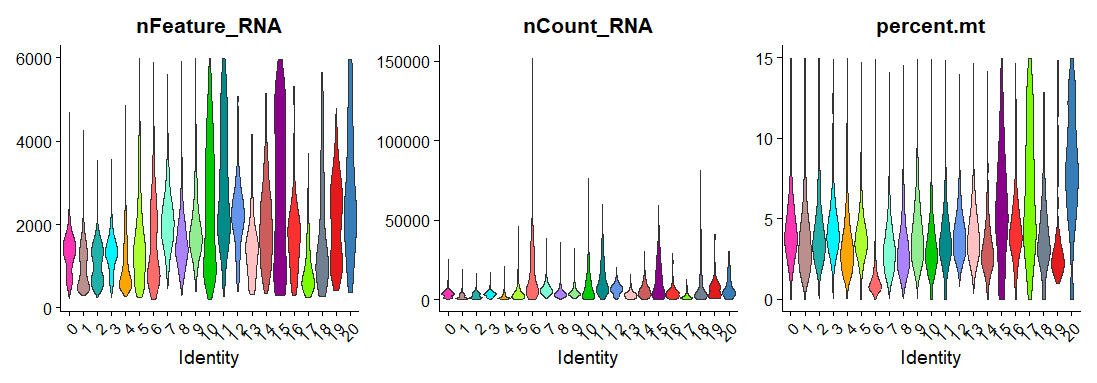


2、Dimensionality reduction clustering


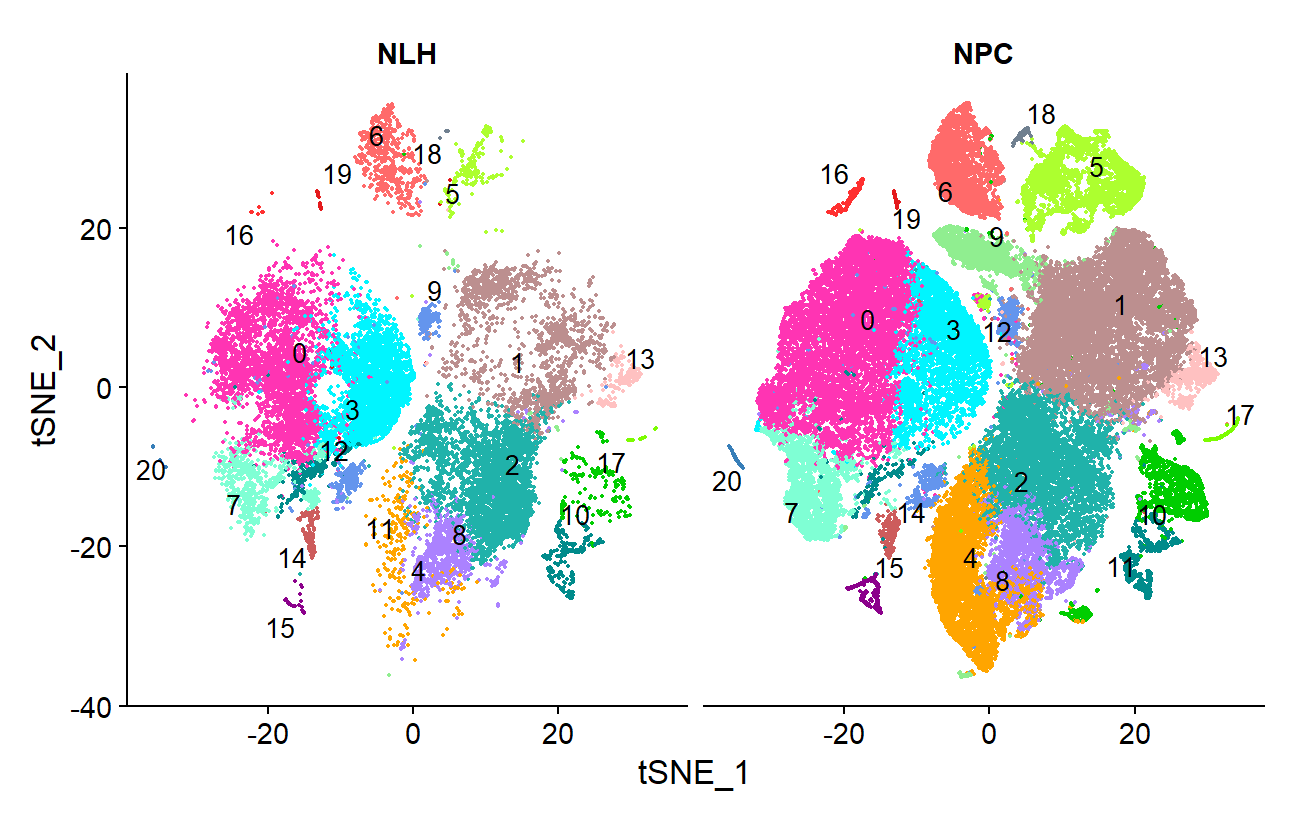


3、markergene used to define cell types


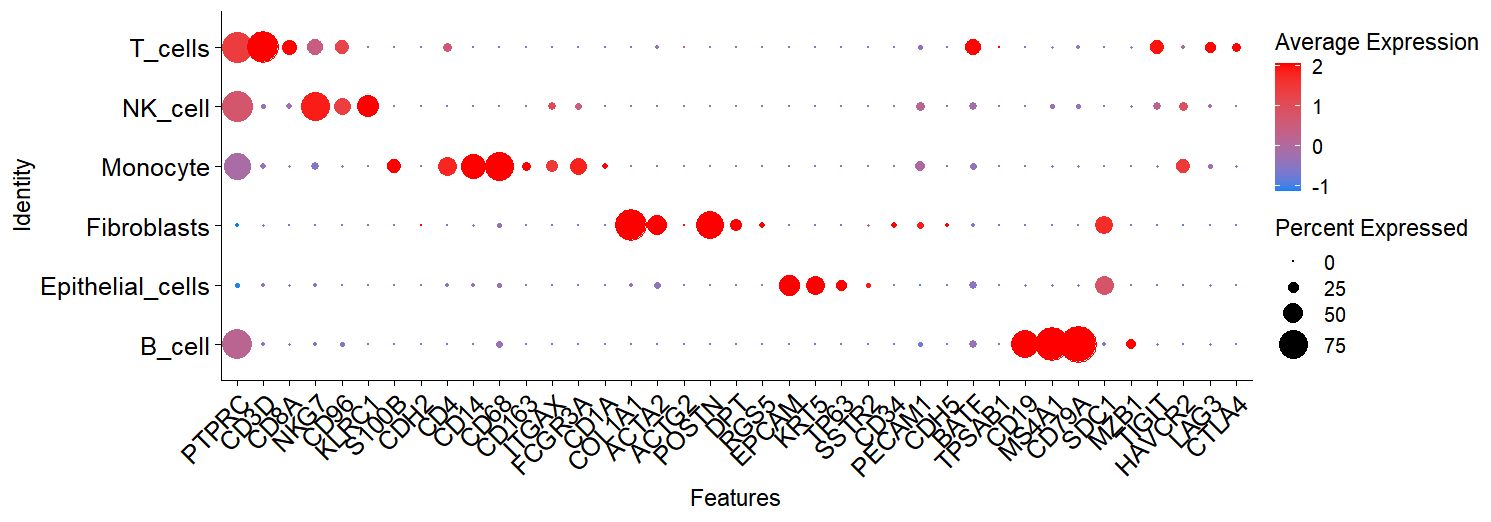


4、different cell type of GSE150825


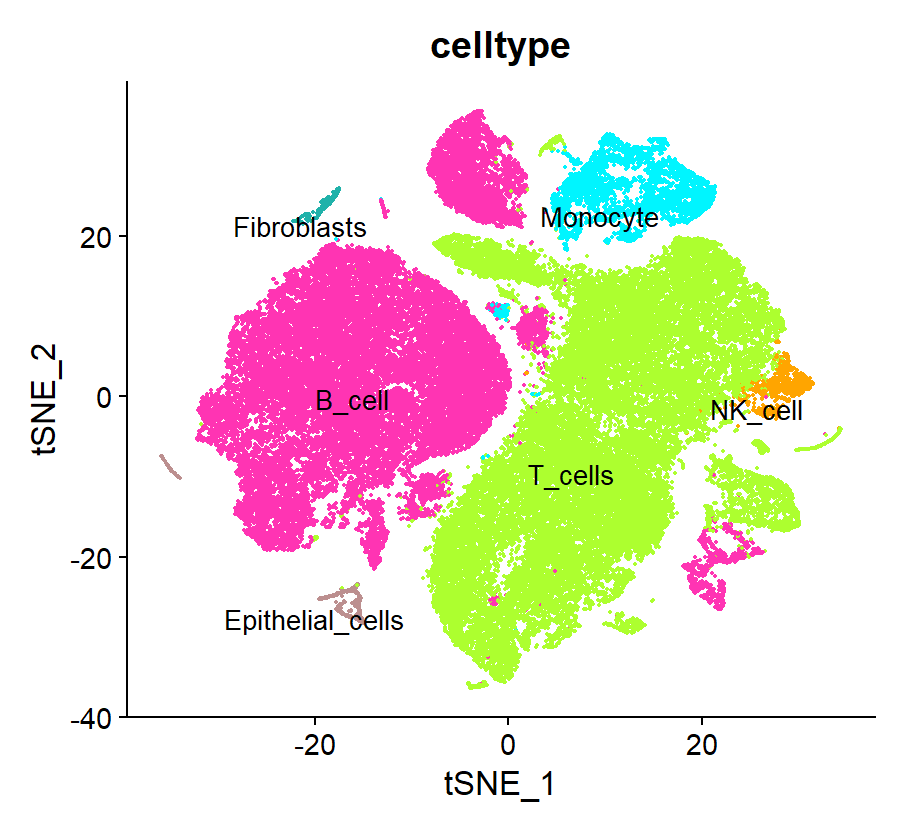


5、exhausted marker of all cell type in GSE150825


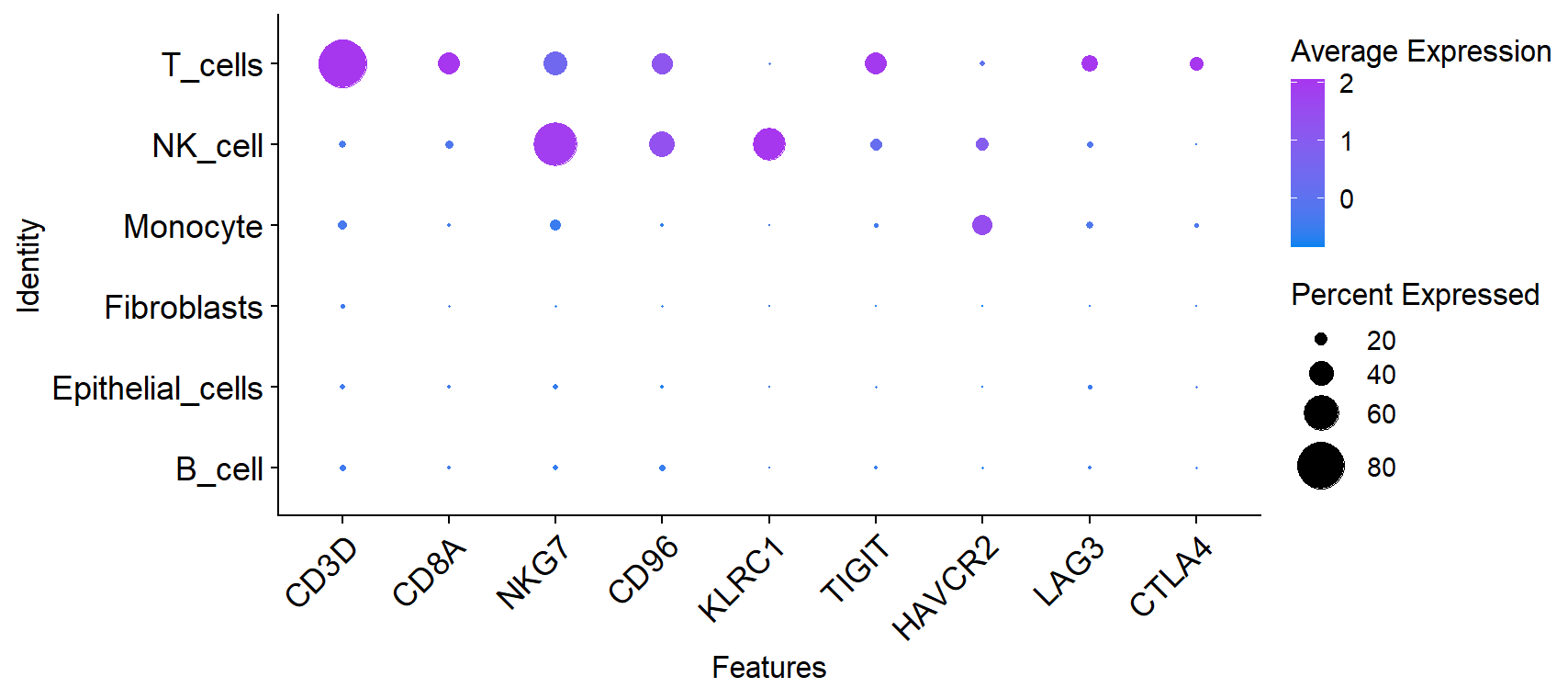


5、different cell type of GSE150825 - split by sample type (NLH :nasopharyngeal lymphatic hyperplasia; NPC: Nasopharyngeal carcinoma)


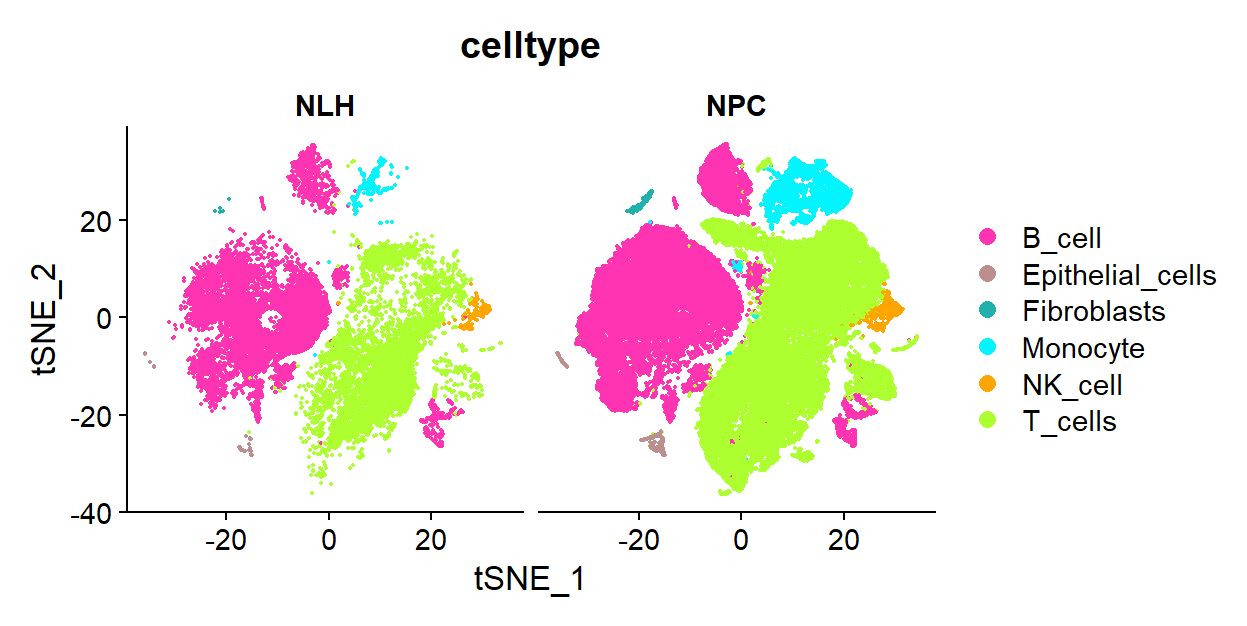


6、NK cell subset（nk1-5）


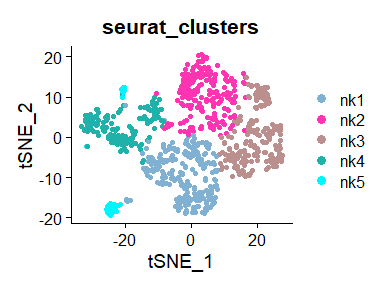


7、NK cell group by sample type


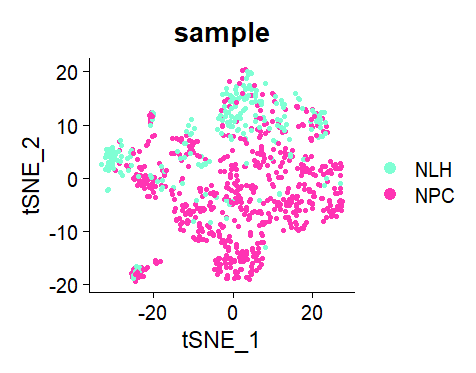


8、expression of nk1-5’s exhausted markers


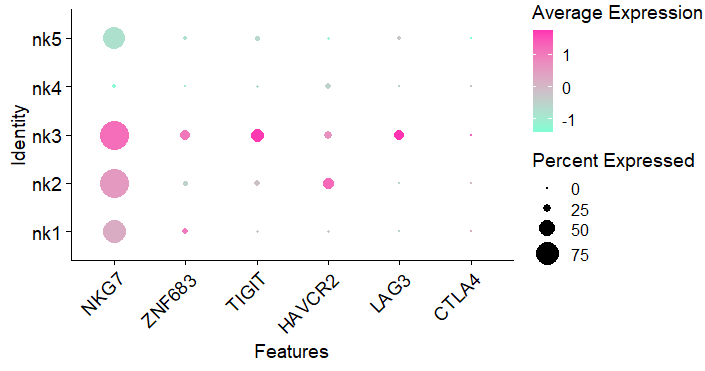

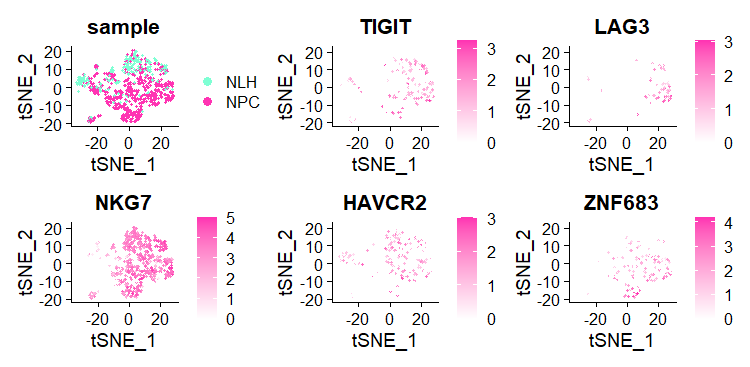

Supplement: Supplementary file 5 — Additional file 5. Datapreprocessing and quality control process for GSE150825 [file 12967_2023_4112_MOESM5_ESM.docx]
